# From macromolecules to electrons—grand challenges in theoretical and computational chemistry

**DOI:** 10.3389/fchem.2013.00006

**Published:** 2013-05-27

**Authors:** Thomas S. Hofer

**Affiliations:** Theoretical Chemistry Division, Center for Chemistry and Biomedicine, Institute of General, Inorganic and Theoretical Chemistry, University of InnsbruckInnsbruck, Austria

Among the many achievements of the twentieth century the accelerating development of microprocessors and their capabilities is one of the most impressive accomplishments, influencing virtually every aspect of daily life. The impact of this new technological resource was (and still is) of particular importance for theoretical approaches in science and engineering. Although many theories (Schrödinger, 1926a,b; Dirac, 1928; Feynman et al., 2010) required for an accurate treatment of quantum systems such as light and matter have been formulated prior to the construction of vacuum tube computers and the invention of the transistor, the applicability of these methodologies is strongly linked to the capacities of the employed computational equipment.

Initially being merely considered as a supplement to experimental work, the steady and consistent development of theoretical approaches significantly extended their accuracy and capabilities. At present many of these disciplines comprise well-established, independent research fields. The computational treatment of chemical systems (Allen and Tildesley, 1990; Szabo and Ostlund, 1996; Levine, 1999; Sadus, 1999; Helgaker et al., 2000; Frenkel and Smit, 2002; Cook, 2005; Dyall, 2007; Reiher and Wolf, 2009; Tuckerman, 2010) is a prominent example of this development. Although modern computational and theoretical chemistry is the result of decades of brilliant research and an impressive wealth of knowledge has been gathered, a number of topics show still as much activity as during the early phases of this discipline. A breakthrough in any of these topics would mark an exceptional milestone and therefore, these questions are considered as grand challenges in the field of theoretical and computational chemistry in the next decades.

Perhaps one of the most prominent challenges in chemistry became known as the protein folding problem (Dill and MacCallum, 2012), i.e., the prediction of the folded structures of peptide and protein systems. While it is tempting to focus on a “sequence-to-structure” relationship, it was argued that such a direct translation does not exist (Ben-Naim, 2013) simply due to the fact that proteins may fold differently when exposed to different chemical environments. Although the protein folding problem may be considered as a purely biochemical topic, it is a highly interdisciplinary field of research, linking biology and biochemistry to fields such as analytical, inorganic, medicinal, organic, physical, pharmaceutical and theoretical chemistry. The latter enables the possibility to investigate the system on a microscopic (i.e., atomistic) level, whereas the vast majority of experimental approaches work in the macroscopic regime.

The most critical aspect determining the accuracy of a computational treatment of chemical systems lies in the description of the atomic interactions. Despite the tremendous capabilities of modern high performance computing facilities, it is still necessary to formulate a compromise between accuracy of results and computational effort. At present pairwise-additive, non-polarizable, empirical potential approaches referred to as molecular mechanical or force field methods (Leach, 2001; Cramer, 2002; Jensen, 2006; Ramachandran et al., 2008) represent the state-of-the-art, but the question whether these approaches are sufficiently accurate has been asked on a regular basis (Hummer et al., 2008; Allison et al., 2011; Beauchamp et al., 2012).

Among the many interatomic forces occurring within such systems, the accurate representation of the solute-solvent interaction is of particular difficulty. While in the past the surrounding solvent was largely regarded as necessary evil when conducting simulation studies (in some cases it was even ignored), an increased awareness of the importance of the solvation is observed in literature in recent years (Levy and Onuchic, 2004; Zaccai, 2004; De Simone et al., 2005; Mamontov and Chu, 2012; Xu et al., 2012). On the other hand the interaction between the biomacromolecule and solvent is of utmost importance, since the resulting intermolecular forces guide the protein into its folded state (Ben-Naim, 2013).

Research focused on an accurate description of biomolecular solvation can be regarded as one grand challenge, which due to the sheer size of proteins and the associated number of molecules involved in the solvation process is by no means a simple task. Since the respective potential models are of empirical nature and experimental data delivers only macroscopic information of the investigated systems, the question arises which data serve as accurate reference on the microscopic level. Naturally, the answer to this lies in the domain of quantum chemistry (Szabo and Ostlund, 1996; Levine, 1999; Helgaker et al., 2000; Cook, 2005; Dyall, 2007; Reiher and Wolf, 2009; Cramer, 2002).

In contrast to empirical models, which rely on parameterized interactions, quantum chemical methods aim to achieve an accurate description of chemical systems by representing the electron density surrounding the nuclei. In this approach nuclei and electrons form the building blocks for all chemical interactions, with Schrödinger's equation constituting the fundamental relation for the energy of the quantum system (Schrödinger, 1926b). Thus, while force fields are typically tailored to a particular class of molecules [e.g., biomolecular systems (Jorgensen and Tirado-Rives, 1988; Cornell et al., 1995; Jorgensen et al., 1996; MacKerell Jr. et al., 2000; Duan et al., 2003; Ponder and Case, 2003; Mackerell, 2004), organic compounds (Schuler et al., 2001), ionic liquids (Borodin, 2009; Maginn, 2009; Dommert et al., 2012), minerals (Cygan et al., 2004), metals (Daw and Baskes, 1984; Finnis, 1984; Stillinger and Weber, 1985), etc.], quantum chemistry offers a general approach applicable to virtually all chemical systems. Modern electronic structure theory formulated in the framework of molecular orbital theory (Szabo and Ostlund, 1996; Levine, 1999; Helgaker et al., 2000; Cook, 2005) and density functional theory (Koch and Holthausen, 2002; Sholl and Steckel, 2009) has become a well-established field of theoretical chemistry.

In general quantum chemical methods are more accurate than empirical potential models, but due to the complexity of this approach the computational effort is also larger by many orders of magnitude, which prevents the application of accurate quantum chemical methods to biochemical macromolecules as of yet. The acceleration of these approaches constitutes an important research topic in theoretical chemistry and can be seen as an important grand challenge. Impressive achievements in linear scaling methods (Goringe et al., 1997; Scuseria, 1999; Goedecker and Scuseria, 2003; Schutz and Manby, 2003; Beer and Ochsenfeld, 2008; Schweizer et al., 2008; Doser et al., 2010; Maurer et al., 2012) have been presented during the last decade and the implementation of fast quantum chemical algorithms in the framework of graphical processing unit (GPU) architectures (Ufimtsev and Martinez, 2008a,b, 2009a,b; Watson et al., 2010; Bao et al., 2011) attracted increased interest, lately.

At this point it might also prove useful to (re-)consider alternative approaches in quantum chemistry, as have been summarized in a recent publication asking: *Solving The Schrödinger Equation: Has Everything Been Tried?* (Popelier, 2011) Explicitly correlated methods (Rychlewski, 2004) and intracule functional theory (Gill, 2011) are just two examples of approaches that are developed aiming at improving the quantum description of atoms and molecules. Recently, the fascinating yet enigmatic property of quantum entanglement characterized (among other properties) via the von Neumann entropy has been suggested to be a promising route to investigate the challenging electron correlation problem (Huang and Kais, 2005; Wang and Kais, 2007; Manzano et al., 2010; Dehesa et al., 2012), i.e., the error introduced by the independent particle approximation to the probability function.

Accuracy consideration and general applicability are not the only advantage of quantum mechanical computations over the use of empirical approaches: the majority of force fields can not (or not adequately) describe the formation and cleavage of chemical bonds. However, in recent years so-called reactive force fields (van Duin et al., 2001; Hofmann et al., 2007; Mahadevan and Garofalini, 2007; Lammers et al., 2008; Knight et al., 2010) have emerged, that are capable to model chemical reactions within a fraction of the computing time required for a QM treatment of the system. Similar as in the case of non-reactive force fields preparameterized potential functions are used, but since the functional forms are far more complex, the parameterization of these reactive approaches is particularly challenging. Currently, reactive force fields for applications in material science and proton transfer dynamics are available and recent activities aim at the extension of these approaches to biomolecular systems (Rahaman et al., 2011). The possibility to model enzyme reactions via an efficient yet accurate molecular mechanical approach is highly desirable and the formulation of reactive force fields for biochemical systems can thus be regarded as a grand challenge as well. Typically data derived from quantum chemical computations serve as reference and benchmark for the empirical force field models, implying that the respective grand challenges are highly interconnected. In addition, new developments in the respective fields will not only impact life sciences but will also be of great benefit for material science oriented research such as the computational treatment of catalysis phenomena (Nørskov, 2000; Frenking, 2005; Bligaard et al., 2009) and investigation of nanosystems (Bichoutskaia, 2011; Varga and Driscoll, 2011).

Nuclear quantum effects (NQEs) are another topic that received increased interest in recent years. NQEs are strongly linked to the computational treatment of hydrogen transfer reactions, which are of critical importance for many chemical disciplines such as catalysis and biochemistry. Due to the low mass of hydrogen atoms, nuclear quantum effects may have a significant impact on the reaction dynamics (Agarwal et al., 2002; Wang et al., 2006), although cases have been reported in which hydrogen atoms exhibit an essentially classical behavior (Tuckerman et al., 1997; Marx et al., 1999). To account for such effects in simulation studies an alternative formulation of quantum mechanics known as path-integral theory (Feynman et al., 2010) is employed. Although a number of established approaches for the execution of path-integral simulations are available today (Cao and Voth, 1994a,b; Jang and Voth, 1999; Craig and Manolopoulos, 2004; Braams and Manolopoulos, 2006), the computation of accurate quantum dynamics from such simulations proved to be a particularly challenging problem. Despite the fact that a number of improved approaches have been reported in recent years (Krilov et al., 2001; Habershon et al., 2007; Paesani and Voth, 2008; Bonella et al., 2010a,b), the computation of accurate long-time quantum correlation functions is still considered as another grand challenge in theoretical chemistry.

Each of these grand challenges is strongly dependent on the development of improved computational facilities. Since it has been estimated that the progress in microprocessor technology will continue throughout the next decades, the formulation of improved approaches addressing the grand challenges will constitute a promising and exciting research activity in the future. It can be expected that theoretical approaches in chemistry assume a similar leading role in chemical science, as observed for theoretical methods in physics during the last century.
